# LDH as an adjuvant makes *Brucella* outer-membrane vesicles and outer-membrane vesicle-associated proteins highly protective in mice

**DOI:** 10.22038/IJBMS.2023.67394.14775

**Published:** 2023

**Authors:** Xiaoyu Deng, Jinke He, Jinfeng Xu, Yueli Wang, Jihai Yi, Huan Zhang, Yong Wang, Zhen Wang, Chuangfu Chen

**Affiliations:** 1 School of Animal Science and Technology, Shihezi University, Shihezi, Xinjiang 832003, China; 2 Collaborative Innovation Center for Prevention and Control of High Incidence Zoonotic Infectious Diseases in Western China, College of Animal Science and Technology, Shihezi University, Shihezi, China; 3 Department of Basic Medicine, Xinjiang Second Medical College, Kelamayi, Xinjiang, China; 4 Wuwei Vocational College, Wuwei, Gansu 733000, China; # These authors contributed equally to this work

**Keywords:** Adjuvant, Brucella, Immunogenicity, Outer-membrane vesicles, Vaccine

## Abstract

**Objective(s):**

Existing *Brucella* vaccines are attenuated and can cause vaccine-associated brucellosis; and these safety concerns have affected their application. Although subunit vaccines have the advantages of safety, efficacy, low cost, and rapid production, they are usually poorly immunogenic and insufficient to trigger persistent immunity. Therefore, we added layered double hydroxide (LDH) as an adjuvant to *Brucella* subunit vaccine formulations to enhance the immune response to the antigen.

**Materials and Methods:**

LDH and Freund’s adjuvant were combined with *Brucella* outer-membrane vesicles (OMVs) and OMV-associated proteins to form a subunit vaccine, respectively. The immunogenicity of LDH as an adjuvant was assessed in BALB/c mice. We examined levels of immunoglobulin G, G1, and G2a (IgG, IgG1, and IgG2a) antibodies (aBs); percentages of Cluster of Differentiation 4-positive (CD4^+^) and CD8^+^ T cells in peripheral-blood lymphocytes; and secretion of cytokines in mouse spleen lymphocytes. Finally, splenic index and splenic bacterial load were assessed via Brucella challenge experiments on mice.

**Results:**

The LDH subunit vaccine also produced high levels of specific aBs in mice compared with Freund’s adjuvant subunit vaccine and induced mainly T-helper 1 cell (T_h_1)-type immune responses. In addition, mice in the LDH subunit vaccine group had significantly lower bacterial loads in their spleens than those in the Freund’s adjuvant subunit vaccine group, and the LDH-OMV vaccine offered a higher level of protection against *Brucella* attack.

**Conclusion:**

LDH as an adjuvant-paired vaccine provided a high level of protection against *Brucella* infection.

## Introduction

*Brucella* spp. is a pathogenic intracellular, gram-negative pathogen that causes brucellosis in humans and many animals, and remains widespread in many countries around the world (1). In domestic and wild animals, *Brucella* infection mainly causes infertility or abortion in females and orchitis, epididymitis, and arthritis in males (2). In humans, in addition to anorexia, headache, myalgia, and back pain, *Brucella* infection produces many nonspecific symptoms such as intermittent fever and associated sweating, chills, malaise, and nausea (3). Currently, vaccination of animals with live attenuated vaccines, such as S19, Rev.1, S2, RB51, and SR82, is an effective measure of controlling brucellosis. However, these live attenuated vaccines still have some drawbacks. For example, the residual virulence of these attenuated vaccines can cause vaccine-associated brucellosis, most cases of which cannot be distinguished from natural infection and vaccination using serologic testing (4). For these reasons, researchers have worked to develop safe and effective subunit vaccines for *Brucella*.

As early as 1966, outer-membrane vesicles (OMVs) were observed in *Escherichia coli* via electron microscopy (5). These OMVs are constitutively released by gram-negative bacteria grown in the log phase, such as *Brucella* spp. (6), *Salmonella* spp. (7), *Vibrio* spp. (8), and *Neisseria* spp. (9). OMVs are spherical vesicles 25–250 nm in size with double membranes that are composed of lipopolysaccharide (LPS), outer-membrane protein (OMP), phospholipids, periplasmic fractions, and cytoplasmic proteins; and contain abundant bacterial-surface antigens (10-12). The size of OMVs makes them readily available for uptake by immune cells, and their natural conformation allows them to present a range of surface-exposed antigens (13). Currently, OMVs are widely used as *in vivo* vaccines. For example, immunization of C57BL/6 mice with *Helicobacter pylori* OMVs induces a T-helper 2 cell (T_h_2)-biased immune response and significantly reduces bacterial load after challenge (14). Inoculating mice with *Acinetobacter baumannii *OMVs provided complete protection against infection (15). Immunizing chickens with avian pathogenic *Escherichia coli* (APEC) OMV completely protected the birds from infection (16). Inoculation of mice with OMVs of* Brucella* 2308 and RB51 induced a significant protective immune response (17). OMVs of different bacteria have shown promising results in the development of subunit vaccines, and therefore they represented an attractive vaccine antigen.

The development of subunit vaccines depends on the choice of those antigens and adjuvants that trigger protective immunity (18, 19). Layered double hydroxide (LDH) is a “sandwich”-shaped, two-dimensional (2D) clay material with a layered crystal structure. LDH resembles alum and has shown high adjuvant properties in biomedical applications (20). As early as 2010, LDH was used as a vaccine neoadjuvant (21); it can effectively deliver antigen to antigen-presenting cells (APCs). LDH antigen significantly promotes the maturation and antigen presentation of dendritic cells (DCs) (22). Later, Chen *et al*. showed that LDH-formulated immunotherapeutic vaccines for infection were effective in promoting the proliferation of memory T cells (23). In both cellular and humoral immunity, LDH exhibits high adjuvant activity (24).

Currently, LDH is commonly used in antiviral and antitumor vaccine formulations, but few studies have been performed on its use in bacterial vaccines (25, 26). In this study, we selected Brucella OMVs, OMP complex, OMP BP26, and OMP10 as immune antigens. These antigens were emulsified with LDH and Freund’s adjuvant to prepare different subunit vaccines, the immune effects of which were compared in BALB/c mice. Finally, we compared the immunity levels of these two adjuvants via challenge experiments in the mice.

## Materials and methods

***Bacterial strains and LDH***

We purchased B. melitensis biotype 3 and B. melitensis strain M5 from the Chinese Center for Disease Control and Prevention (Beijing, China). B. melitensis biotype 3 and M5 were cultured in trypticase soy broth (TSB; Difco Labs, Inc., Detroit, MI, USA). We obtained LDH from Prof. Wenyi Gu of The University of Queensland, Australia. All materials containing live Brucella were handled in a biosafety level 3 (BSL-3) laboratory facility.

***Experimental animals***

We purchased Female BALB/c mice from the Experimental Animal Center of Zhengzhou University (Henan, China). All experimental procedures complied with animal care regulations and usage guidelines of China (No. GB149258-2010). 

***Preparation of OMVs***

OMVs were extracted according to the method previously described by Pollak *et al.* (27). Briefly, we collected the bacteria by centrifugation after culturing of *B.* *melitensis *biotype 3 to an optical density at a wavelength of 600 nm (OD_600_) value of 0.1, and then resuspended them in *Brucella* nutrient-deficient medium for 24 hr. The bacterial suspension was centrifuged at 10,000 *g* for 30 min, and the supernatant was collected. We filtered the supernatant through a 0.22-μm filter (Merck KGaA, Darmstadt, Germany), coated it onto tryptic soy agar (TSA) and incubated it at 37 ^°^C for 72 hr to assess its sterility. Next, we ultracentrifuged the sterile supernatant at 100,000 g for 2 hr at 4 ^°^C. The precipitate was washed twice with sterile phosphate-buffered saline (PBS), and then the precipitate (the OMVs) was resuspended in sterile PBS. We determined the total protein concentration of OMVs using a Bicinchoninic Acid (BCA) Protein Kit (Thermo Fisher Scientific, Waltham, MA, USA) and stored it at -20 ^°^C until use.

***Preparation of OMP and OMV-related proteins***

OMP complexes were extracted according to the method previously described by Verstreate *et al.* (28). Briefly, we collected* B.* *melitensis* biotype 3 and sonicated it in an ice bath for 45 min. The sonicated suspension was centrifuged at 10,000 rpm for 30 min, after which the supernatant was collected and added to 0.1 mol/l pre-cooled Na_2_CO_3_ (pH, 11.0) with slow stirring for 1 hr, and then ultracentrifuged at 30,000 rpm for 1 hr. We collected the precipitate, suspended it in 50 mmol/l Tris-Cl (pH, 7.5), and ultracentrifuged the suspension at 30,000 rpm for 1 hr. The precipitate was collected again and fully dissolved in lysis solution (7 mol/l urea, 4% 3-[(3-cholamidopropyl) dimethylammonio]-1-propanesulfonate [CHAPS], 1% dithiothreitol [DTT], protease inhibitor). The suspension was *B. melitensis* OMP complexes. The OMPs BP26 and OMP10 were prepared using *E. coli* expression vectors as described previously (29).

***Western blot analysis***

To evaluate the expression of OMPs, we separated purified BP26 and OMP10 using 12% sodium dodecyl sulfate polyacrylamide gel electrophoresis (SDS-PAGE) and transferred them onto a 0.25-μm nitrocellulose (NC) membrane (MilliporeSigma, Burlington, MA, USA). The NC membrane was closed with 5% skimmed milk powder at 37 ^°^C for 2 hr, washed three times with tris-buffered saline+Polysorbate 20 (TBST), and incubated with a 1:500 dilution of *Brucella*-positive serum (from goats) at 37 ^°^C for 1 hr. Next, we washed the membrane three times in PBS with a low-concentration detergent solution (PBST) and incubated it with 1:1000 horseradish peroxide (HRP)-coupled rabbit anti-sheep immunoglobulin G (IgG) antibody (aB; Abcam, Cambridge, UK) at 37 ^°^C for 1 hr. Detection was performed using a HRP-3,3′-diaminobenzidine (DAB) substrate chromogenic kit (TIANGEN, Beijing, China).

***Preparation of vaccines***

We produced subunit vaccines using two different adjuvants: LDH, which has been shown to be an effective adjuvant to enhance vaccine immunity, and Freund’s adjuvant. LDH has been shown to capture antigens *in situ*, enhance immune cell antigen presentation, and induce individualized immune responses (20, 22). In addition, it has been demonstrated effective as an adjuvant in inactivated foot-and-mouth disease and antitumor vaccines (25, 26). We prepared LDH by the method described by Shi *et al.* (20, 30). The LDH subunit vaccine groups were prepared by emulsifying OMV, OMP, BP26, and OMP10 with LDH at a ratio of 1:16 (Figure S1). In addition, we emulsified OMV, OMP, BP26, and OMP10 with Freund’s adjuvant (Sigma Germany, Munich, Germany) at a ratio of 1:1 to prepare the Freund’s adjuvant subunit vaccine group. A total of eight subunit vaccines were prepared.

***Animal experiments***

For immunization, the optimal dose of each antigen was considered as described in a previous report (31-33). In short, we randomly divided a total of 135 female BALB/c mice, 6 weeks old, and weighing 20.6±0.3 g (mean±standard error of the mean [SEM]), into nine groups (n=15). Mice were inoculated with each of the eight subunit vaccines via intraperitoneal (IP) injection at a dose of 30 μg/each. We used a phosphate-buffered saline (PBS) group as a control; 15 BALB/c mice were inoculated IP with PBS at a dose of 100 μl/each. Four weeks after immunization, mice received boosters of the same dose of vaccine or PBS. We collected blood samples from mouse tail veins at 0, 7, 14, 21, 28, 35, and 42 days post-vaccination (dpv) and collected the serum via centrifugation at 4 ^°^C to detect IgG, IgG1, and IgG2a aBs using an indirect enzyme-linked immunosorbent assay (ELISA) method as described previously (34). 

***Measurement of CD4*** ^+^ ***and CD8*** ^+^ ***T lymphocytes in peripheral blood***

We determined counts of CD4^+^ and CD8^+^ T lymphocytes in the peripheral blood of mice by CD4^+^ and CD8^+^ aBs (BioLegend CNS, Inc., San Diego, CA, USA) as described previously (29). Briefly, after 35 days of immunization, whole blood was collected from mice tail veins using an anticoagulation tube. We added 2 μl of CD4^+^ and CD8^+^ aBs to 100 μl of the whole-blood sample, shook and mixed it, and then incubated the mixture for 30 min in the dark at room temperature. Next, we added 2 ml erythrocyte lysis solution, mixed well, and lysed for 8 min in the dark until the cell suspension was clear and transparent. Finally, we added 1 ml PBS to wash the cells and measured the percentages of CD4^+^ and CD8^+^ via flow cytometry (FCM; Roche Life Science, Basel, Switzerland). Ten mice were tested in each group.

***Measurement of cytokine production by lymphocytes***

To determine cytokine concentrations in lymphocytes, we selected six mice per group for euthanasia 2 weeks after booster immunizations, and then we isolated splenic lymphocytes by aseptic removal of the spleen. Lymphocytes were stimulated with inactivated *Brucella* biotype 3 (multiplicity of infection [MOI], 10:1), using PBS as a negative control and concanavalin A (50 μg/ml) as a positive control. Stimulation was performed with inactivated *Brucella* biotype 3 at a MOI of 10:1. We determined lymphocyte concentrations of interleukins-4 and -10 (IL-4, IL-10), interferon gamma (IFN-γ), and tumor necrosis factor alpha (TNF-α) using cytokine ELISA kits (R&D Systems , Inc., Minneapolis, MN, USA) as previously described (35).

***Mouse challenge experiments***

At 42 dpv, each mouse was inoculated i.p.y with 100 μl of 2.0×107 colony-forming units (CFU)/ml *B.* *melitensis* M5. Fourteen days post-challenge, six mice per each group were selected for euthanasia, their spleens were removed under aseptic conditions, and spleen weight was compared with BW by measuring the splenic index of the mice. We completely ground mouse spleens to homogenization using a tissue homogenizer; then we serially diluted 100 μl of tissue homogenate 10×, coated it on TSA, and incubated it at 37 ^°^C in a 5% CO_2_ incubator for 3-5 days for colony counting. Each experiment was repeated three times. Experimental results were analyzed using SPSS software v17.0 (IBM Corp., Armonk, NY, USA) and expressed as log_10_ CFU. The formula we used, as described previously (36), was as follows:

protective units=PBS group spleen log_10_CFU-immunized group spleen Log_10_CFU.

***Statistical analysis***

Statistical analysis was performed using a two-tailed Student’s t test, Fisher’s exact test, or the Mann-Whitney *U* test in SPSS. *P<0.0*5 was considered statistically significant. We plotted dose-response curves using GraphPad Prism software v7.0 (GraphPad Software, Inc., San Diego, CA, USA).

## Results

***Identification of OMVs, OMP, BP26, and OMP10***

**Figure 1:**
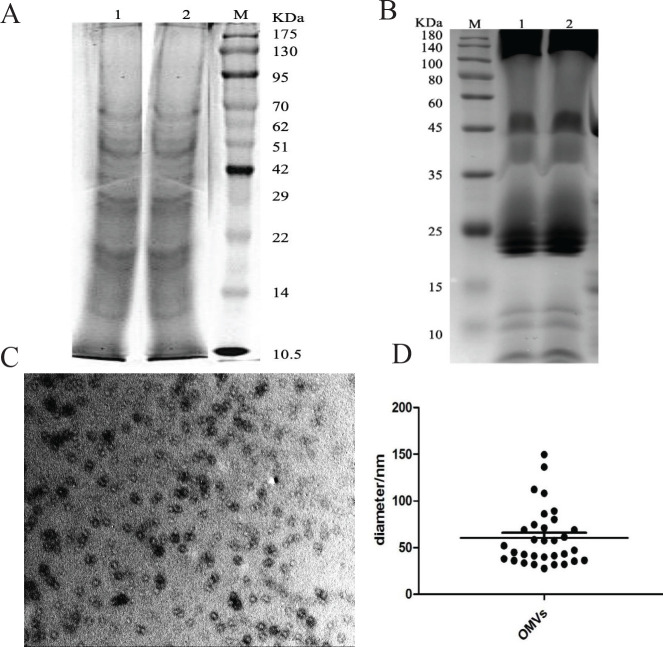
Identification of outer membrane vesicles (OMVs) and outer membrane protein complexes (OMP)

**Figure 2:**
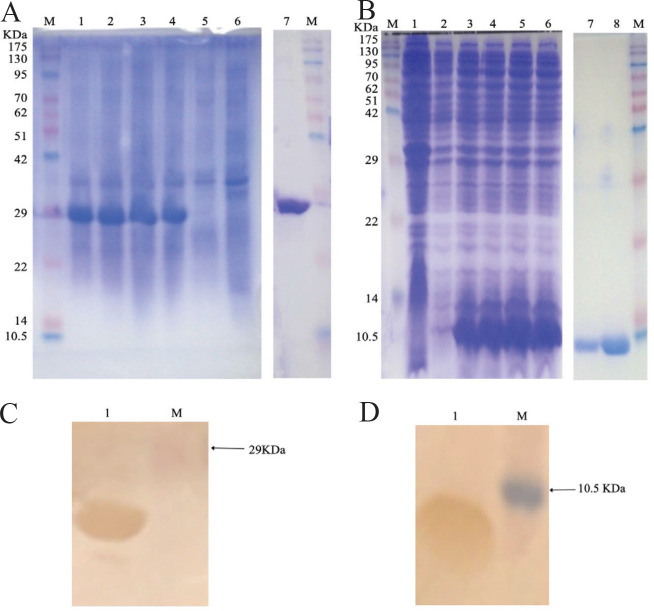
Identification of outer membrane protein BP26 and outer membrane protein OMP10

We examined OMVs and the OMPs BP26 and OMP10) of *Brucella* via transmission electron microscopy (TEM) and SDS-PAGE. The results showed that OMVs of *B.* *melitensis* biotype 3 were 60.5 nm±30 nm in diameter, mainly concentrated around 58 nm (Figure 1C-D). SDS-PAGE results showed that the OMVs contained proteins that were mainly 10-70 KDa (Figure 1A), and the OMPs contained proteins that were mainly between the ranges of 20–30 KDa and 100–180 KDa (Figure 1B). We successfully obtained BP26 protein 28 KDa in size and OMP10 protein 10.4 KDa in size using the *E. coli* expression system (Figure 2A-B). In addition, we successfully verified the ability of these two proteins to react specifically with *Brucella*-positive sera via protein immunoblotting (Figure 2C-D).

***Assessing effectiveness of immunization by measuring serum antibody levels***

**Figure 3:**
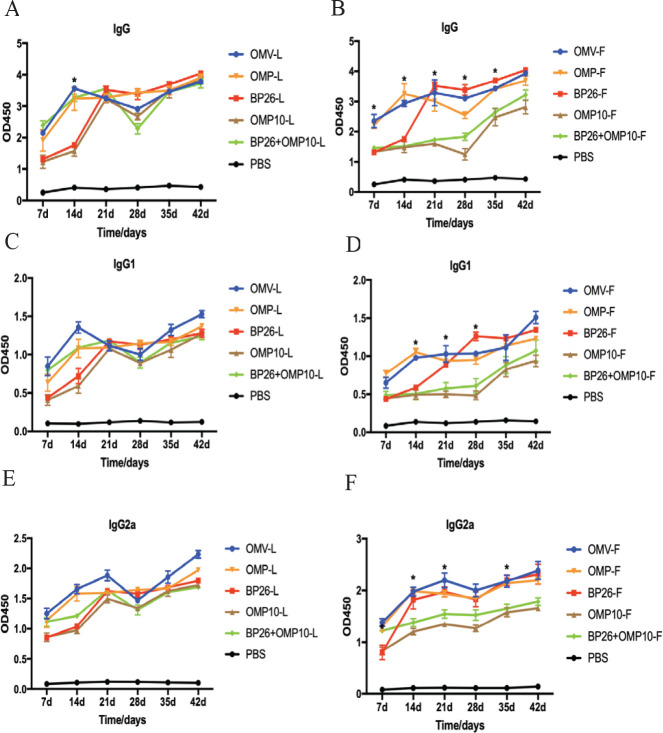
Changes in serum antibody levels in mice after post-vaccination

We evaluated the immunization effect of the vaccine via indirect ELISA detection of aB levels, with higher OD values indicating higher aB levels. The results showed that aB levels continued to increase after the first immunization, started to decrease after 21 days, and started to increase again 28 days after booster immunization (Figure 3A-3B). At 21-35 dpv, IgG aB levels were significantly higher in the OMV-F, OMP-F, and BP26-F (Freund’s adjuvant) groups than in the BP26+OMP10-F and OMP10-F groups (*P<0.0*5; Figure 3B), but we saw no significant difference between the LDH adjuvant groups (Figure 3A). This indicated that the LDH adjuvant could produce higher levels of IgG aBs in the OMV, OMP, BP26, OMP10, and BP26+OMP10 groups, while Freund’s adjuvant could produce high levels of IgG aBs only in the OMV, OMP, and BP26 groups. In addition, the BP26+OMP10-L and OMP10-L (LDH adjuvant) groups produced significantly higher levels of IgG aBs than the BP26+OMP10-F and OMP10-F (Freund’s adjuvant) groups (Figure S2). There was no significant difference between IgG1 and IgG2a levels in the LDH adjuvant groups *versus* the Freund’s adjuvant groups (*P*>0.05), but IgG2a levels were higher than IgG1 levels in both types of groups (Figure 3C-F), indicating a shift toward a T_h_1-type response after vaccination. In conclusion, the subunit vaccine in the LDH adjuvant groups was more effective in stimulating IgG aB production in mice than that in the Freund’s adjuvant groups.

***Comparison of immune effects by measuring lymphocyte subpopulation ratios***

Levels of CD4^+^ and CD8^+^ in T lymphocytes can directly reflect an animal’s immune function. We compared the immune effect of the vaccines in the LDH and Freund’s adjuvant groups by the ratio of CD4^+^ to CD8^+^ in peripheral-blood T lymphocytes. The percentage of CD4^+^ cells was significantly increased in the LDH adjuvant group compared with PBS-immunized mice (*P<0.0*5), but there was no significant increase in that of CD8+ cells (Table 1). In addition, the percentage of CD4^+^ cells was significantly increased in the Freund’s adjuvant (OMV-F, OMP-F, BP26-F, and BP26+OMP10-F) groups (*P<0.0*5), whereas the increase in CD4^+^ cells was not significant in the OMP10-F group (Table 2). As in the LDH adjuvant group, there was no significant increase in the percentage of CD8^+^ cells in the Freund’s adjuvant group (Table 2). Overall, we found no significant difference in CD4^+^ percentage between the Freund’s and LDH adjuvant groups (*P>0.0*5), indicating that the LDH adjuvant vaccine was as effective as the Freund’s adjuvant vaccine in promoting CD4^+^ T-lymphocyte levels.

***Comparison of immune effects by measuring cytokine secretion by lymphocytes***

**Figure 4:**
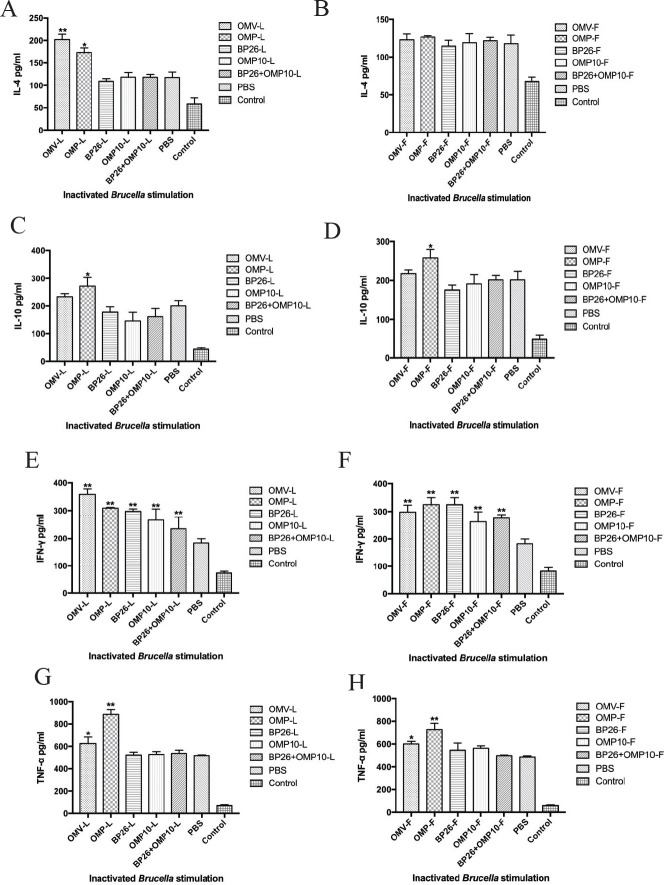
Cytokines produced by spleen cells from mice immunized with either the Freund’s adjuvant group or the LDH adjuvant group

Cytokine production by activated T cells is an indicator of T_h_ response type. We evaluated cytokine secretion via ELISA after stimulation of splenic lymphocytes by inactivated *Brucella* (36). The results showed that lymphocytes from mice in the Freund’s adjuvant group could not induce significant IL-4 secretion compared with the PBS control group (*P>0.0*5; Figure 4B). However, in the OMV-L and OMP-L (LDH adjuvant) groups, lymphocytes could induce secretion of significantly higher amounts of IL-4 (*P<0.0*5; Figure 4A). Both the OMV-L (LDH adjuvant) and the OMP-F (Freund’s adjuvant) groups demonstrated secretion of significantly higher amounts of IL-10 compared with the PBS control group (*P<0.0*5), with no significant difference between the two (*P>0.0*5; Figure 4C-D). Lymphocytes in both the Freund’s and LDH adjuvant groups induced significantly higher amounts of IFN-γ secretion than those in the PBS control group (*P<0.0*1), and the OMV-L group demonstrated significantly higher IFN-γ secretion than the OMV-F group (*P<0.0*5; Figure 4E-F). Both the OMV-L and OMP-L (LDH adjuvant) groups and the OMV-F and OMP-F (Freund’s adjuvant) groups showed a significantly higher amount of TNF-α secretion than the PBS control group (*P<0.0*5), and lymphocytes in the OMP-L group induced significantly higher TNF-α secretion than those in the OMP-F group (*P<0.0*5; Figure 4G-H). In conclusion, the LDH adjuvant vaccine effectively promoted cytokine secretion by lymphocytes to a greater degree than the Freund’s adjuvant vaccine.

***Protective effects of subunit vaccines with different adjuvants in mice***

**Figure 5:**
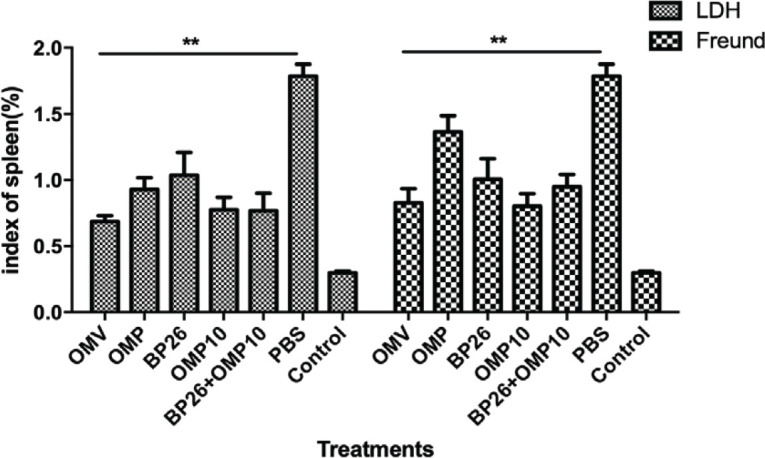
Analysis of spleen index in mice after brucella M5 challenge

We quantified levels of vaccine protection by measuring the splenic index and the number of bacteria in the spleen (log10 CFU) 14 days after *Brucella *M5 challenge. The results showed that the splenic indices of mice in the LDH and Freund’s adjuvant groups were significantly lower than that in the PBS control group (*P<0.0*1; Figure 5), and the splenic index of the OMP-L LDH adjuvant group was significantly lower than that of the OMP-F Freund’s adjuvant group (*P<0.0*5; Figure 5). Splenic bacterial counts showed that the immune-induced protection level of all vaccines in the Freund’s and LDH adjuvant groups was significantly higher than that in the PBS control group (*P<0.0*5; Tables 3 and 4), indicating that the vaccines in both adjuvant groups were significantly protective in mice. The level of protection against attack was 2.58 log in the OMV-L group and 1.82 log in the OMV-F group, but there was no significant difference between the two groups (*P>0.0*5; Tables 3 and 4). In addition, we saw no significant difference between the OMP-L, BP26-L, OMP10-L, and BP26+OMP10-L groups and the OMP-F, BP26-F, OMP10-F, and BP26+OMP10-F groups (*P>0.0*5; Tables 3 and 4). These results suggested that the LDH adjuvant group experienced a significant protective effect against *Brucella* M5 infection in mice compared with the Freund’s adjuvant group.

(A) Identification of outer membrane vesicles (OMVs) of *Brucella melitensis* biotype 3 by SDS-PAGE. (B) Identification of outer membrane protein complexes (OMP) of *B. melitensis* biotype 3 by SDS-PAGE. (C) Identification of outer membrane vesicles (OMVs) by transmission electron microscopy. (D) Characterization of the particle size of outer membrane vesicles (OMVs)

(A) Identification of outer membrane protein BP26 of *Brucella melitensis* biotype 3 by SDS-PAGE. (B) Identification of outer membrane protein OMP10 of *B. melitensis* biotype 3 by SDS-PAGE. (C) Identification of outer membrane protein BP26 of *B. melitensis* biotype 3 by immunoblotting. (D) Identification of outer membrane protein OMP10 of *B. melitensis* biotype 3 by immunoblotting

**Table 1:** Analysis of peripheral blood T-lymphocyte subsets after vaccination of mice with LDH adjuvant group

Group	OMV-L	OMP-L	BP26-L	OMP10-L	BP26+OMP10-L	PBS
CD4+	47.57±3.51^c^	39.71±2.13^b^	35.50±2.89^b^	36.88±0.96^b^	37.34±2.11^b^	30.99±1.34^a^
CD8+	16.46±0.55^d^	16.34±2.53^d^	15.53±3.11^d^	15.96±2.34^d^	15.87±1.32^d^	15.09±1.01^d^

After 35 days of post-vaccination, peripheral blood from mice immunized with OMV-L, OMP-L, BP26-L, OMP10-L, BP26+OMP10-L, and PBS was collected and labeled with FITC-CD4 antibody and PE-CD8 antibody, and the ratio of CD4^+^ and CD8^+^ cells was measured by flow cytometry. Ten mice were tested in each group, and all data are expressed as Mean±SD. Different superscript lowercase letters indicate significant differences between different groups of mice (*P<*0.05)

Mice were vaccinated with subunit vaccines prepared by emulsifying OMV, OMP, BP26, and OMP10 with LDH adjuvant and Freund’s adjuvant, and sera were collected at 7, 14, 21, 28, 35, and 42 days post-vaccination. (A) (B) IgG antibody levels were measured by indirect ELISA. (C) (D) IgG1 antibody levels were measured by indirect ELISA. (E) (F) IgG2a antibody levels were measured by indirect ELISA. Antibodies were tested in five mice per group and expressed as Mean±SD

**Table 2:** Analysis of peripheral blood T-lymphocyte subsets after vaccination of mice with Freund’s adjuvant group

Group	OMV-F	OMP-F	BP26-F	OMP10-F	BP26+OMP10-F	PBS
CD4+	44.26±2.17^c^	40.90±1.73^c^	37.86±1.46^c^	33.56±0.95^a^	39.29±1.92^c^	30.99±1.37^a^
CD8+	16.91±2.58^d^	16.16±2.43^d^	15.30±3.17^d^	15.42±1.64^d^	16.40±0.83^d^	14.94±1.25^d^

Thirty-five days post-vaccination, peripheral blood from mice immunized with OMV-F, OMP-F, BP26-F, OMP10-F, BP26+OMP10-F, and PBS was collected and labeled with FITC-CD4 antibody and PE-CD8 antibody, and the ratio of CD4^+^ and CD8^+^ cells was measured by flow cytometry. Ten mice were tested in each group, and all data are expressed as Mean±SD. Different superscript lowercase letters indicate significant differences between different groups of mice (*P<*0.05)

Two weeks after booster immunization, splenic lymphocytes from mice were isolated and stimulated with inactivated B. melitensis biotype 3, and the concentrations of IL-4 in the LDH adjuvant group (A) and Freund’s adjuvant groups (B) were measured by ELISA kits. The concentrations of IL-10 in the LDH adjuvant group (C) and Freund’s adjuvant groups (D) were measured by ELISA kits. The concentrations of IFN-γ in the LDH adjuvant group (E) and Freund’s adjuvant groups (F) were measured by ELISA kits. The concentrations of TNF-α in the LDH adjuvant group (G) and Freund’s adjuvant groups (H) were measured by ELISA kits. Six mice were tested in each group, and all data are expressed as Mean±SD. *P<0.05 and **P<0.01 indicate significant differences

The Freund’s adjuvant and LDH adjuvant groups were booster immunized for two weeks and challenged with *Brucella abortus* M5, and spleen weight was compared with bodyweight by measuring the spleen index of the mice 14 days after the challenge

**Table 3:** Protection against challenge with *Brucella* M5 after immunization with LDH adjuvant group vaccine

Vaccine	LogCFU/spleen	Log
	(Mean±SD)	protection
OMV-L	3.04±0.33^a^	2.58
OMP-L	4.39±0.37^b^	1.23
BP26-L	3.28±0.40^a^	2.34
OMP10-L	3.86±0.56^b^	1.76
BP26+OMP10-L	4.21±0.41^b^	1.41
PBS	5.62±0.17^c^	0

Six mice were tested in each group, and all data are expressed as Mean±SD. Different superscript lowercase letters indicate significant differences between different groups of mice (*P<*0.05)

**Table 4:** Protection against challenge with *Brucella* M5 after immunization with Freund’s adjuvant group vaccine

Vaccine	LogCFU/spleen	Log
	(Mean±SD)	protection
OMV-F	3.80±0.69^a^	1.82
OMP-F	4.20±0.37^a^	1.42
BP26-F	3.84±0.70^a^	1.78
OMP10-F	3.49±0.28^c^	2.13
BP26+OMP10-F	4.03±0.29^a^	1.59
PBS	5.71±0.16^c^	0

Six mice were tested in each group, and all data are expressed as Mean±SD. Different superscript lowercase letters indicate significant differences between different groups of mice (*P<*0.05)

## Discussion

Currently, there is no *Brucella* vaccine for use in humans because all* Brucella* vaccines are attenuated and can therefore cause human infection. *Brucella* subunit vaccines have become a hot topic of research (18). Subunit vaccines have the advantages of high safety, low cost, and rapid production, but recombinant protein-based subunit vaccines are usually poorly immunogenic and insufficient for triggering a durable immune response. Therefore, adjuvants must be added to subunit vaccine formulations to enhance immune response to the antigen (37). In this study, we chose layered double hydroxide (LDH), which has high adjuvant activity. Previous studies have found that subunit vaccines prepared from a single bacterial protein usually do not protect the organism from *Brucella *infection (38-40). Herein, we used *Brucella* outer-membrane vesicles (OMVs) and outer-membrane protein (OMPs) complexes as immune antigens paired, respectively, with LDH adjuvant and Freund’s adjuvant. Levels of humoral immunity elicited by the vaccines in mice, levels of cellular immunity, and the vaccines’ protective properties in mice were evaluated and compared with those of mice immunized with *Brucella *OMPs BP26 and OMP10.

 T_h_1-type responses play an important role in immune clearance of intracellular pathogens, and IFN-γ is essential for control of *Brucella* infection (36, 41). Our results found that both the LDH adjuvant and Freund’s adjuvant vaccines produced significant levels of IFN-γ. Moreover, the LDH adjuvant-paired OMV vaccine could produce higher levels of IFN-γ in splenic lymphocytes of mice than the Freund’s adjuvant vaccine (Figure 4E–F). Mouse experiments have shown that TNF-α plays an important role in the control of *Brucella *infection in mice (42). In this study, splenic lymphocytes from mice immunized with LDH adjuvant (OMV-L and OMP-L) and Freund’s adjuvant (OMV-F and OMP-F) vaccines produced high levels of TNF-α on re-exposure to inactivated *Brucella*, but there was no significant difference between the two adjuvant groups (Figure 4G–H). In addition, the LDH adjuvant (OMV-L and OMP-L) vaccine produced high levels of IL-4 (T_h_2 cytokine) in the splenic lymphocytes of immunized mice (Figure 4A-B), while no significant changes were observed in the Freund’s adjuvant group. We also observed IL-10 (T_h_2 cytokine) production in mice immunized with the OMP-F and OMP-L vaccines (Figure 4C-D). IL-10 is thought to be an immunosuppressive factor that inhibits macrophage function to increase intracellular survival of *Brucella* (43), but previous studies have shown that IL-10 is induced in mice after *Brucella* vaccination but does not decrease the vaccine’s level of protection in mice (44). We further analyzed IgG, IgG1, and IgG2a aB production and found that all three immunoglobulins increased in a time-dependent manner, indicating that mice exhibited T_h_1 and T_h_2 immune responses after vaccine immunization. In addition, IgG2a levels were higher than IgG1 levels (Figure 3), but it is important to note that IgG1 and IgG2a levels detected using indirect ELISA depend on the affinity of aB levels (36). Therefore, we detected the percentages of CD4^+^ and CD8^+^ T cells in mouse peripheral-blood lymphocytes. The results showed that both the LDH and Freund’s adjuvant vaccines could induce significant levels of CD4^+^ T lymphocytes (*P<0.0*5; Tables 1 and 2) but had no effect on CD8^+^ T cells. Researchers reported that T_h_1 CD4^+^ T cells play an important role in the control of *Brucella* infection, but bacterial clearance by CD8^+^ T cells during primary infection importance can be neglected (41). Our results demonstrated the ability of the LDH adjuvant vaccine to produce significantly higher specific aBs and induce a T_h_1-type immune response in mice.

 Overall, OMV and OMP vaccines paired with LDH adjuvants showed better immunity in mice than did Freund’s adjuvant vaccines. Previous studies reported that OMV-vaccinated mice exhibited high levels of protection against* B.* *melitensis* 16 M challenge (45). Our study showed that immunization of mice with LDH and Freund’s adjuvant vaccines resulted in a significant reduction of bacterial load in the spleens of the mice. In addition, the OMV-L (LDH adjuvant) vaccine showed a higher level of protection against attack than the OMV-F (Freund’s adjuvant) vaccine (Tables 3 and 4). These results suggested that LDH adjuvant-paired OMV and OMP vaccines provided significant protection against *Brucella* M5 infection in mice. So far, we have used only *Brucella attenuata* strains in challenges to initially detect adjuvant effects. Further studies are needed to determine whether our vaccines are protective against strong strains of *Brucella*.

## Conclusion

LDH may be a good adjuvant for the development of *Brucella* subunit vaccines. This adjuvant-paired vaccine provided a high level of protection against *Brucella* M5 infection. In conclusion, our data will provide a new adjuvant platform for the development of *Brucella* subunit vaccines.

## Data availability statement

The datasets presented in this study can be found in online repositories. The names of the repository/repositories and accession number(s) can be found in the article/Supplementary Material.

## Ethics statement

All animals in the study were used in accordance with the recommendations of National Standards for Laboratory Animals of the People’s Republic of China (GB149258–2010). The experimental protocols used in the animal studies were approved by the Shihezi State University Institutional Animal Care and Use Committee. 

## Authors’ Contributions

CC, ZW, and WW conceived and designed the experiments, and JH and JX performed the experiments. XD and JH carried out revision of the manuscript. XD, YW, JY, and HZ analyzed the data and drafted the manuscript. All authors contributed to the article and approved the submitted version.

## Conflicts of Interest

The authors have no conflicts of interest to declare.
